# Intelligent Systems for Inorganic Nanomaterial Synthesis

**DOI:** 10.3390/nano15080631

**Published:** 2025-04-21

**Authors:** Chang’en Han, Xinghua Dong, Wang Zhang, Xiaoxia Huang, Linji Gong, Chunjian Su

**Affiliations:** 1 College of Mechanical and Electronic Engineering, Shandong University of Science and Technology, Qingdao 266590, China; 202282050006@sdust.edu.cn; 2 GBA Research Innovation Institute for Nanotechnology, Guangzhou 510700, China; dongxh@hc.cannano.cn (X.D.); or zhangwang1316@163.com (W.Z.); huangxiaoxia@cannano.cn (X.H.); 3 Guangzhou Guangna Huichuan Technology Co., Ltd., Guangzhou 510700, China; 4 School of Biomedical Science and Engineering, South China University of Technology, Guangzhou 511442, China

**Keywords:** inorganic nanomaterials, intelligent synthesis, artificial intelligence, nanomanufacturing

## Abstract

Inorganic nanomaterials are pivotal foundational materials driving traditional industries’ transformation and emerging sectors’ evolution. However, their industrial application is hindered by the limitations of conventional synthesis methods, including poor batch stability, scaling challenges, and complex quality control requirements. This review systematically examines strategies for constructing automated synthesis systems to enhance the production efficiency of inorganic nanomaterials. Methodologies encompassing hardware architecture design, software algorithm optimization, and artificial intelligence (AI)-enabled intelligent process control are analyzed. Case studies on quantum dots and gold nanoparticles demonstrate the enhanced efficiency of closed-loop synthesis systems and their machine learning-enabled autonomous optimization of process parameters. The study highlights the critical role of automation, intelligent technologies, and human–machine collaboration in elucidating synthesis mechanisms. Current challenges in cross-scale mechanistic modeling, high-throughput experimental integration, and standardized database development are discussed. Finally, the prospects of AI-driven synthesis systems are envisioned, emphasizing their potential to accelerate novel material discovery and revolutionize nanomanufacturing paradigms within the framework of AI-plus initiatives.

## 1. Introduction

By its fundamental, cross-cutting, and leading characteristics, nanotechnology has deeply penetrated key fields such as information, energy, medicine, and manufacturing, promoting technological innovation and industrial upgrading. It has become a transformative and strategic technology for which significant countries and economies compete.

The rapid development of nanotechnology and industry has put forward higher requirements for the large-scale and high-quality preparation of nanomaterials. Although traditional synthesis methods have achieved stage-by-stage progress in the preparation of typical nanomaterials such as quantum dots (QDs), gold nanoparticles (AuNPs), and silica (SiO_2_), they still face bottlenecks such as low reproducibility, difficulties in macroscopic preparation, and insufficient quality control (e.g., particle size uniformity, dispersion, and structural stability). It is difficult to satisfy the urgent needs of downstream industries for consistent material properties and scaled-up supply, which systematically restricts the wide application of nanomaterials.

The design, preparation, and performance regulation of nanomaterials are key development directions in nanoscience. Research on the design of new structures, the accurate measurement of intrinsic physical properties, and large-scale precise preparation technologies of nanomaterials have become a strategic requirement for breaking through the performance bottlenecks of traditional materials and giving birth to emerging industries. Therefore, exploring new synthesis paradigms with high efficiency, precision, and reproducibility has become a key breakthrough to promoting nanomaterials from the laboratory to industrialization.

In recent years, the rise in “AI+” technology has injected innovative power into nanomaterial synthesis. Therefore, we introduce the concept of intelligent synthesis. Intelligent synthesis is an artificial intelligence technology centered on machine learning and deep learning, which integrates automated experimental apparatus with closed-loop feedback systems to achieve material optimization and autonomous exploration in synthesis methods. Furthermore, the synthesis technology of nanomaterials is gradually evolving along the development direction of automation–autonomy–intelligent synthesis. It is progressively establishing a new paradigm of intelligent synthesis based on “data-driven + intelligent optimization” [1,2,3,4,5,6,7]. This paradigm is achieved by integrating high-throughput experimental data acquisition, synthetic parameter modeling and prediction, and dynamic process optimization. It significantly shortens the exploration cycle of the ideal material “structure-efficacy” relationship and addresses the issues of resource waste and low reproducibility in traditional trial-and-error methods.

**Figure 1:**
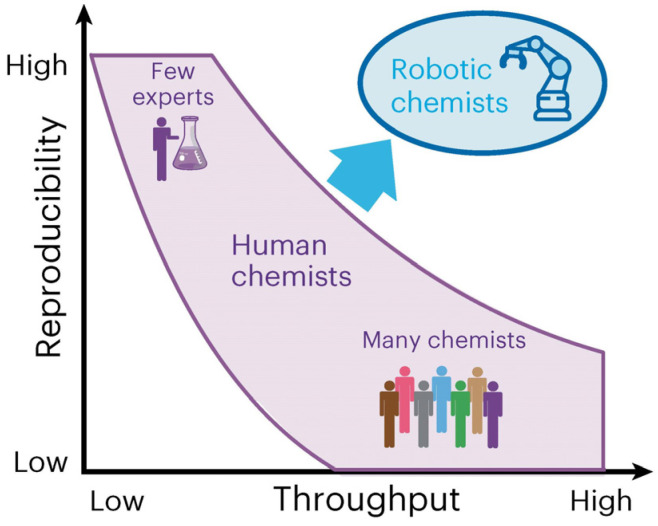
Compared to human experimentalists, robotic chemists can achieve both high reproducibility and throughput simultaneously. Adapted with permission from ref. [12], copyright 2024, the authors.

With the rapid development of Digital Chemistry [8,9], the intelligent synthesis system has evolved into a comprehensive framework encompassing hardware automation, algorithmic intelligence, and human–machine collaboration. Firstly, the automated reaction device enables parametric control, real-time monitoring, and feedback regulation of the synthesis process. Secondly, machine learning algorithms autonomously optimize reaction pathways and predict the structure–efficacy relationship of materials by uncovering hidden patterns within multidimensional data. Moreover, these algorithms can further predict unknown material properties, expanding the scope of intelligent material discovery [10]. This seamless integration of software and hardware has gradually given rise to the concept of the robotic chemist [11], enhancing synthesis efficiency, stability, and reproducibility in product quality control (Figure 1) [12] and paving the way for the exploration of novel nanomaterials, ultimately driving a paradigm shift in synthesis.

Currently, several breakthroughs have emerged in the field of intelligent synthesis of inorganic nanomaterials. This paper focuses on how to build an intelligent synthesis system for inorganic nanomaterials, systematically outlines the latest development trends in the field of intelligent synthesis of inorganic nanomaterials, and focuses on the design of automated hardware systems, the optimization of software systems and intelligent synthesis methods, and optimization strategies with the assistance of AI. In artificial intelligence-assisted nanomaterial synthesis, analysis, and summarization are conducted from the perspective of the entire chemical research process. This dramatically facilitates interdisciplinary personnel to quickly get started and understand the field, allowing them to combine their strengths to conduct appropriate interdisciplinary research. In addition, the key role of machine learning in synthesis parameter optimization, process modeling, and inverse design, as well as its impact on the intelligent synthesis process, is discussed using the intelligent synthesis of several typical inorganic nanomaterials, such as QDs and AuNPs, as case studies. Finally, recommendations are made to address the challenges of standardized databases, human–machine collaboration, and interdisciplinary integration in the field of intelligent synthesis of inorganic nanomaterials, and the opportunities to promote the development of smart nanomanufacturing under the background of “AI+” are also envisioned. This paper aims to provide theoretical references for the systematic development of intelligent synthesis technology and help the precise synthesis of nanomaterials and their industrial applications.

## 2. Design and Optimization of Intelligent Synthesis Systems for Inorganic Nanomaterials

**Figure 2:**
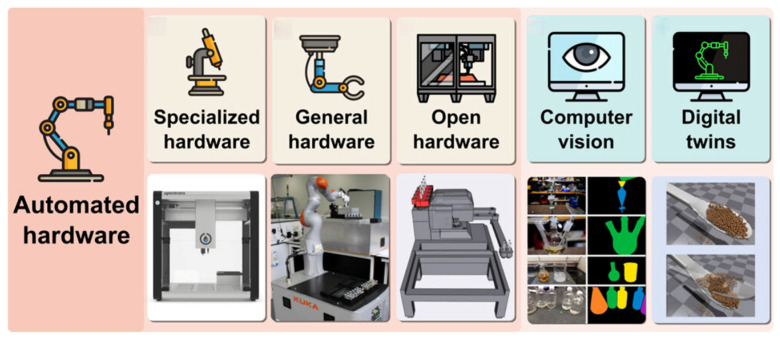
Example of hardware system for intelligent synthesis of nanomaterials. Adapted with permission from ref. [6], copyright 2024, the authors.

In the intelligent synthesis of inorganic nanomaterials, the automated hardware system is the core component of the closed-loop control system (Figure 2), which covers the key aspects of accurate data acquisition, the in situ characterization of materials, and the regulation of synthesis parameters [6]. In terms of hardware design, it is necessary to regulate reaction conditions accurately during the experimental process to meet the high standards of nanomaterial synthesis. Since the synthesis of nanomaterials usually involves a variety of reagents and requires a large number of reagents and consumables, and the variables in the process of reaction parameters are interrelated and have complex influences, the traditional manual operation requires a large amount of workforce and material resources, which is challenging for meeting the demand for efficient, accurate, and reproducible synthesis. Therefore, promoting the automated and autonomous synthesis of nanomaterials is extremely important. Automated synthesis refers to synthesis methods controlled by machines or computers without autonomous decision-making capabilities.

In contrast, autonomous synthesis refers to systems that possess specific adaptive decision-making abilities based on automated synthesis. The vision of the automated and autonomous synthesis of inorganic nanomaterials enhances the efficiency and accuracy of experiments. It reduces human errors and resource consumption, representing an inevitable trend in the future development of nanomaterial synthesis.

### 2.1. Design of Automated Hardware Systems

#### 2.1.1. Design of Automation Hardware System Based on Microfluidic Technology

It is of great importance to apply automated hardware systems, represented by microfluidic technology, in the automated synthesis of inorganic nanomaterials. Microfluidic technology enables the efficient control of the reaction process on a microscopic scale [13]. It extends the synthesis space and makes the synthesis process predictable and reproducible. This technology enables efficient high-throughput preparation of multifunctional nanoparticles while significantly reducing reagent consumption, making microfluidics an ideal tool for exploring the synthesis methods and parameter space of various inorganic nanomaterials [14].

In 2013, Lohse et al. developed a millifluidic reactor for the synthesis of AuNPs [15], which is not only capable of high-throughput, gram-scale preparation of AuNPs, it is also capable of fine-tuning the prepared gold nanorods and realizing the precise control of the aspect ratio of gold nanorods; moreover, all the parts of this reactor can be assembled by purchasing them by themselves. To efficiently characterize AuNPs in real-time and for quality control, the reactor integrates ultraviolet–visible (UV-Vis) absorption spectroscopy and tangential flow filtration. The reactor is compatible with a wide range of gold nanoparticle synthesis methods and has ports for functionality expansion and enhancement upgrades, providing researchers with greater flexibility to explore new synthesis pathways and optimize existing methods.

Automated microfluidic platform technology has become both a crucial tool for optimizing reaction parameters and a powerful approach for gaining deeper insights into the complex mechanisms of nanocrystal (NC) nucleation and growth. In 2015, Abolhasani et al. reported an automated biphasic mini-platform based on a polytetrafluoroethylene (PTFE) reactor [16], which eliminates the residence time limitations imposed by the continuous-flow strategy by controlling the oscillatory motions of the droplets. It can efficiently perform high-throughput in situ studies of solution-phase semiconductor NCs. During the experimental process, droplet formation and in situ absorption spectroscopy data acquisition are automatically controlled by computer scripts, thus realizing the precise control of parameters and reproducibility of the synthesis process. Thanks to the in situ characterization technique, the platform can monitor the state of QDs in the flow system in real-time, revealing the kinetic mechanism of the nucleation and growth stages of colloidal NCs [17].

This oscillating microprocessor is suitable for screening and optimizing the synthesis of other semiconductor NCs. It can also be used for the synthesis study of multiphase nanomaterials, significantly improving the efficiency of nanomaterials research and development. For example, by integrating an automated microfluidic reactor with an online optical detection system, the reaction process information can be collected in real-time and efficiently, and the reaction conditions for the synthesis of perovskite NCs can be rapidly screened [18]. Analyzing the obtained experimental data further verifies the fundamental theories of nanocrystal nucleation and growth and facilitates the development of algorithms for optimizing QD synthesis conditions [19].

#### 2.1.2. Design of Robot-Assisted Automated Hardware Systems

**Figure 3:**
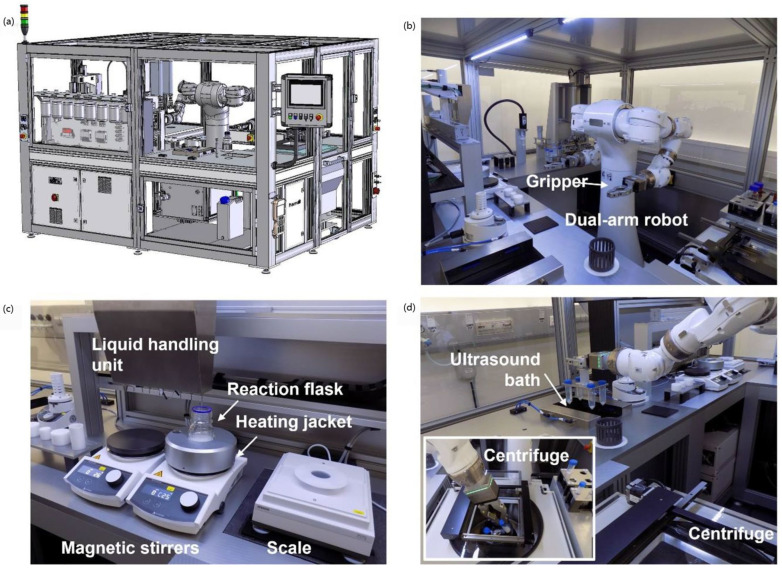
Robot-based plant for automated production of nanoparticles and main components. (**a**) Computer-aided design model showing installation of dual-arm robot in tailored housing made from aluminum profiles. Housing is closed and can be accessed through specific ports that facilitate maintenance and supply with materials. Human–machine interface allows user to control system. (**b**) Dual-arm robot can interact with (**c**) liquid handling station and (**d**) automated centrifuge. Adapted with permission from ref. [20], copyright 2023, the authors.

As automated synthesis systems for inorganic nanomaterials move toward modular design, the core module connecting the reaction equipment will be a robotic system with multiple functions and easy operation. For example, Figure 3 shows an automated synthesis system based on a dual-arm robot [20], which establishes an automated process for the reproducible synthesis of nanoparticles, and its modular design provides a more convenient interface for the non-robotics researcher to drive the robot to complete the synthesis according to the preset experimental steps. The system enables experimenters to obtain higher quality products in a shorter period, and the system can take on a workload that would be difficult for a human to accomplish and maintain an efficient and stable operation, thus significantly improving the synthesis efficiency.

In the validation case, the robotic system converted a traditional manual synthesis protocol for SiO_2_ nanoparticles with a particle size of about 200 nm into an automated process optimized and tailored to the designed experimental requirements while benchmarking both manual and automated synthesis. The results show that the robotic system exhibits excellent efficiency and good reproducibility in SiO_2_ nanoparticle synthesis by comparing different experimental parameters and product quality, significantly reducing labor and time costs. Due to the modular design concept, the system integrates standard laboratory equipment for routine wet chemistry experimental steps such as mixing and centrifugation and is highly scalable. The laboratory can flexibly add different functional modules according to individual synthesis needs, enabling this robot system to achieve more customized functions. The modular dual-arm robotic system offers a highly flexible solution for inorganic nanoparticle synthesis, enhancing process adaptability and enabling the efficient synthesis of typical inorganic nanomaterials.

**Figure 4:**
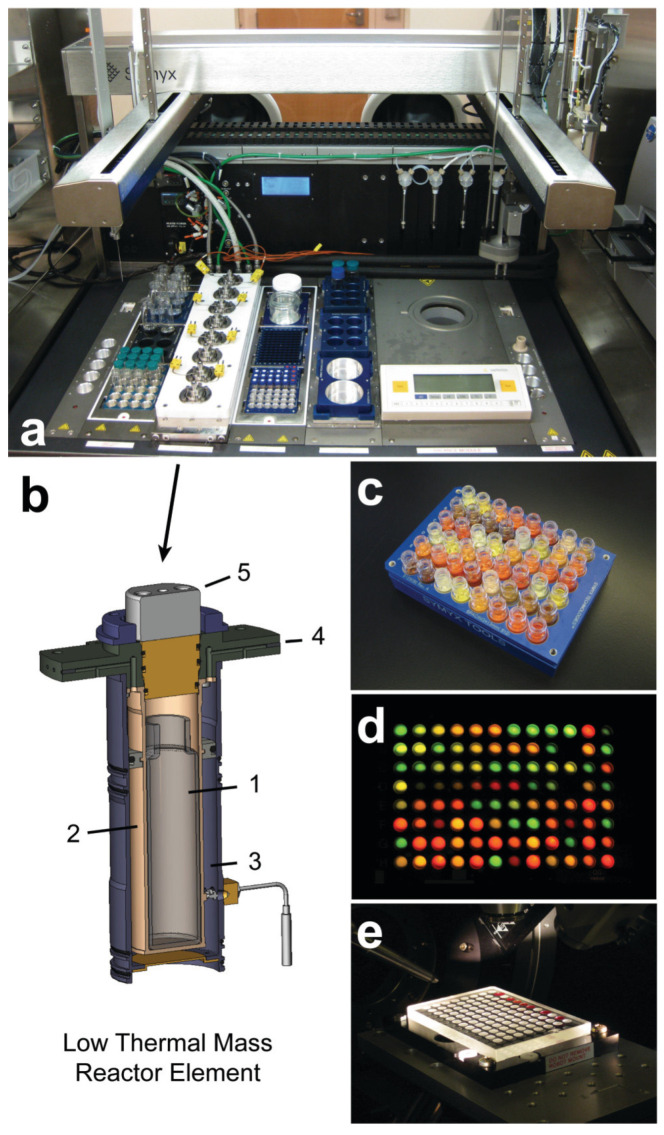
Workstation for automated nanomaterial synthesis. (**a**) Robotic deck featuring two liquid-dispensing robotic arms and eight-element low-thermal mass reactor (LTMR) array for high-temperature nanocrystal synthesis. (**b**) Cross section of LTMR element. A 40 mL glass vial (1) is inserted intothe heated reactor cell (2), which can be cooled rapidly by nitrogen flow into the cooling shroud (3). Reactive gases can be injected through inlets (4), and the reactor can be accessed through configurable caps (5) that allow for pressurization or vacuum purging. (**c**) CdSe nanocrystal aliquots were sampled using robotic arms. (**d**) Photoluminescence of CdSe and CdTe nanocrystal aliquots in 96-well quartz microplate. (**e**) Ninety-six-well glass X-ray diffraction plate for high-throughput X-ray diffraction. Adapted with permission from ref. [21], copyright 2010, American Chemical Society.

In 2010, Chan et al. proposed an automated workstation based on the Symyx Technologies core module platform [21], shown in Figure 4, to synthesize QDs efficiently. The workstation is feature-rich and highly integrated, equipped with a liquid-handling robot for solution transfer, a heated needle for dispensing molten surfactants, a bottle fixture for manipulating solid objects, and an automated metrology balance for recording sample mass. The workstation is also equipped with a customized deck assembly containing an array of eight high-temperature reactors (up to 300 °C), with each reactor element featuring independent temperature control and digitally adjustable magnetic stirring to rapidly and precisely ramp up and down to the desired synthesis temperature. To minimize cross-contamination, the glass reaction vessels in the elements are disposable and consumable. Taking the synthesis of CdSe NCs as an example, the researcher achieved a minimal coefficient of variation from batch to batch, demonstrating the workstation’s precise control of multiple reaction parameters. By integrating high-throughput optical and diffraction characterization techniques, the researcher could efficiently map the multidimensional parameter space and achieve optimization of the CdSe nanocrystal property parameters. In addition, to ensure that the entire automated synthesis process was carried out in a water- and oxygen-free environment, all equipment except the diffractometer was placed in a nitrogen-filled glove box.

**Figure 5:**
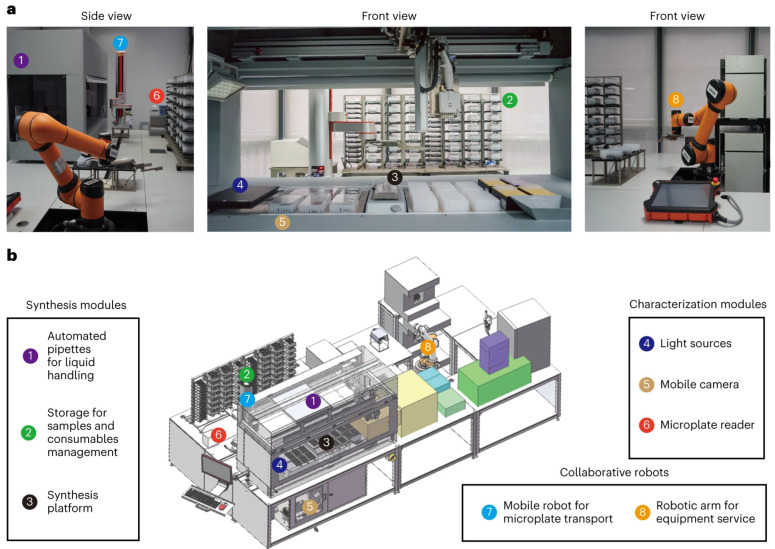
Robotic platform for nanocrystal synthesis and characterization. (**a**) Side view and front view photographs and (**b**) schematic illustration. Adapted with permission from ref. [22], copyright 2023, the authors.

In recent years, Jiang’s group built an automated nanoparticle synthesis system in the laboratory [22], as shown in Figure 5, which integrates an automated pipette, sample and consumable storage, a synthesis platform, a light source, a color-ultra-sensitive mobile camera, and a microtiter plate reader, and works in tandem with a mobile robot and a robotic arm, which to some extent realizes robot-assisted high-throughput synthesis of nanomaterials and in situ characterization. In addition, the system is equipped with a corresponding literature mining module, based on which the synthesis process is optimized by intelligent algorithms to form a closed-loop feedback mechanism, thus realizing the automated and efficient synthesis of nanomaterials.

Automated synthesis systems are gradually transitioning to modular design to better adapt to the increasingly complex synthesis needs and realize flexible synthesis. Since performing multistep automated synthesis expansion still faces some difficulties in a single customized automated synthesis system, manual intervention or reconfiguration is often required to accomplish different reaction protocols. For this reason, Angelone et al. developed a Chemputer synthesis robot capable of performing multiple reactions [23]. To enhance the flexibility and efficiency of this robotic system, the team also developed generalized modular hardware that can be automated and controlled by a single software system. The system successfully performed about 8500 operations and accessed 17 different reactions while reusing only 10 unique modules for 22 different steps. The chemical reaction requirements for different material synthesis needs can be met with minor modifications to the system hardware. By continuously expanding the library of reaction modules, the synthesis robot can support even more diverse chemical reactions and meet the high standards researchers demand for automated material synthesis. By expanding the modularization concept and the library of automated synthesis modules, the hardware, software, and other technologies are continuously integrated to gradually form an open and unified standard synthesis platform, which improves the reproducibility of experimental processes and results and significantly enhances the innovation efficiency of the researcher [24].

With the increasing demand for automation in nanomaterial synthesis, building an automated synthesis system based on modularization has become an important future research direction for the intelligent synthesis of inorganic nanomaterials. Establishing a comprehensive modular standard system for automated synthesis is essential to meet the increasingly diverse demands of chemical reactions and to enhance resource utilization efficiency [6]. This system classifies different reactions according to the experimental conditions they require. It formulates the corresponding modular hardware standards, which improves the system’s extensibility and operability and reduces the technological thresholds for researchers to build an automated synthesis system [25]. The system can also be used as a basis for the development and implementation of automated synthesis systems, which can be used for the synthesis of inorganic nanomaterials. At the same time, hardware standardization can reduce errors caused by hardware incompatibility in the synthesis process, thus improving the reliability of the research results. With the rapid development of robotics technology and the continuous improvement of algorithm capabilities, the automation level of the nanomaterial synthesis process is expected to be further enhanced, thus achieving a breakthrough in autonomy [26,27].

### 2.2. Optimization of Automated Synthesis Equipment

#### 2.2.1. In Situ Characterization Equipment and Optimization

During the synthesis of inorganic nanomaterials, commonly used in situ characterization methods include transmission electron microscopy (TEM), XRD, UV-Vis absorption spectroscopy, and fluorescence spectroscopy. These methods provide real-time feedback on nanoparticles’ morphology, dimensions, crystal structure, elemental composition, and optical properties.

For example, the most representative TEM characterization method can provide nanoscale high-resolution images to reveal key information such as nanoparticle morphology and defects. However, the imaging data volume of traditional TEM is vast and complex, and there is an urgent need to optimize its automated analysis software. By adopting optimized automated scanning transmission electron microscopy (STEM) data acquisition and analysis techniques, nanoparticles’ physical and compositional information can be acquired at high resolution on a micrometer-length scale [28]. Meanwhile, an intelligent system built based on machine learning algorithms can control and make imaging adjustments through various detector feedback, significantly improving the equipment’s analysis efficiency and accuracy [29,30].

Microscopic analysis has not yet been fully automated due to the lack of uniformity in low-level instrument control standards for TEM and hardware and software compatibility issues. For this reason, Olszta et al. designed an automated measurement system that combines low-level instrument communication with task classification based on few-sample machine learning [31]. The system enables easy programming operations for the end user by creating a low-level component abstraction layer from multiple manufacturers. The application of this system will dramatically improve the efficiency of microanalysis, and its task-oriented, high-throughput statistical analysis-based approach will significantly enhance the accuracy of the characterization results compared with traditional automated analysis methods by continuously optimizing the microanalysis process through system feedback.

**Figure 6:**
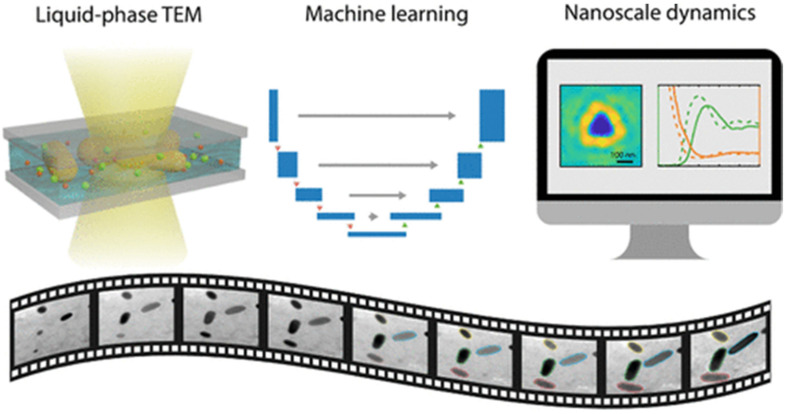
Machine learning to reveal nanoparticle dynamics from liquid-phase TEM videos. Adapted with permission from ref. [32], copyright 2020, the authors.

With the popularization of liquid-phase TEM in materials analysis, researchers can observe the dynamic processes of nanomaterials at the nanoscale in real-time. However, traditional automated analysis methods are deficient in the efficiency of dynamic data extraction. To reduce the impact of high noise and spatial heterogeneity in the video on the detection results, Yao et al. combined liquid-phase TEM imaging with a customized analysis framework based on a machine learning model of the U-Net neural network (Figure 6), which improves the ability of the device to analyze the dynamics of various reactions and phase transitions [32]. U-Net is a fully convolutional neural network based on convolutional neural networks, which employs an encoder–decoder structure and skip connections to achieve high-precision pixel-level segmentation. It is widely used in biomedical image segmentation [33]. The U-Net model’s robust image segmentation capabilities can effectively overcome noise interference and spatial heterogeneity of particle intensity in situations with low signal-to-noise ratio and high background fluctuations. This enables it to efficiently and accurately identify and segment the shapes and boundaries of nanoparticles.

Machine learning-based optimization of liquid-phase TEM enhances analysis efficiency. It supports in-depth studies of nanoscale dynamic behavior, laying a crucial technical foundation for developing automated and intelligent nanomaterial synthesis systems.

#### 2.2.2. Real-Time Data Collection and Feedback System

The “data flywheel” effect generated by automated synthesis systems is crucial [4,34]. It represents the initial step in optimizing inorganic nanomaterial synthesis and provides a data foundation for subsequent process improvements. Therefore, precise control of reaction conditions and real-time in situ monitoring are essential in nanomaterials’ continuous and automated synthesis processes.

Fiber optic ultraviolet–visible spectral detection technology has been widely employed for the online monitoring of the synthesis of noble metal NCs in liquid phases [34]. For instance, Guda et al. utilized an improved Latin hypercube sampling method to adjust reaction parameters [35], coupled with in situ UV-Vis spectroscopy, to obtain critical optical data in real-time during the nanocrystal synthesis process, thereby forming a spectral dataset that facilitates an in-depth investigation into the formation, morphology, and growth processes of nanoparticles. Furthermore, high-throughput semi-automated photoluminescence spectroscopy has also been employed for the in situ monitoring of the liquid-phase synthesis of Cu_1−x_Ag_x_InS_y_Se_1−x_ (CAISSe) QDs as well as CAISSe/ZnS core/shell structured QDs [36]. During the experimental process, the size information of the NCs is acquired by measuring the localized surface plasmon resonance (LSPR) spectra of the noble metal NCs. Additionally, the small outer diameter, low optical loss, and high flexibility of fiber optics provide an effective interface for the assembly of online monitoring devices, enabling integration with computational models further to optimize the continuous synthesis methods of colloidal NCs.

It is crucial to integrate online detection systems into the automated framework to ensure the precise control of automated synthesis systems and the closed-loop optimization of reaction processes. For instance, microfluidic technology has demonstrated significant effectiveness and vast application potential in the automated multidimensional screening of reaction parameters through in situ optical characterization [14,19]. By incorporating detection modules, microfluidic devices can simultaneously acquire absorption and fluorescence data on timescales ranging from milliseconds to seconds [19]. Furthermore, the axial and rotational movement of heating rods allows for monitoring the optical properties of QDs at various locations along the reaction pathway. Additionally, the design of inline optical measurements facilitates the extraction of fluorescence spectra and the assessment of absorbance.

In summary, online monitoring systems, especially optical spectral characterization, significantly enhance the controllability and precision of the synthesis process by capturing key data during the reaction in real-time. Moreover, reaction parameters can be further optimized when appropriate sampling methods are combined with computational models.

#### 2.2.3. Intelligent Operation and Precise Recognition

**Figure 7:**
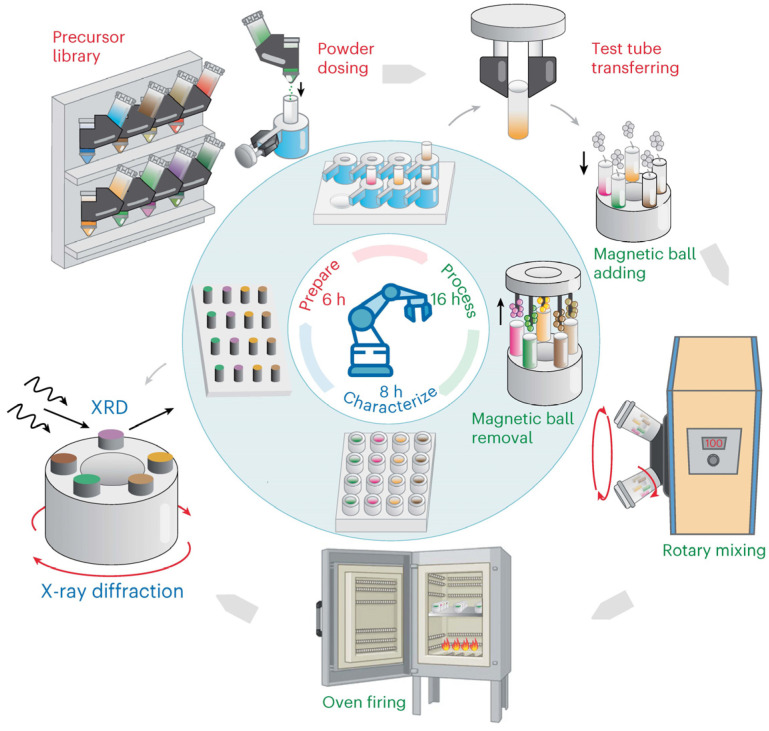
A robot-enabled inorganic material synthesis workflow—from powder precursor preparation to ball milling, to oven firing and to XRD characterization of reaction products. Adapted with permission from ref. [12], copyright 2024, the authors.

Robotics based on various types of sensing functions is becoming more widespread to increase the degree of automation in the synthesis of nanomaterials. In addition, the rapid development of AI in recent years has dramatically expanded the range of applications and experimental capabilities of machine chemists and experimental robots (Figure 7) [12].

To solve the problem of weighing powder samples in synthetic experiments, Kadokawa et al. proposed a simulation-to-reality-based transfer learning method with domain randomization (DR) technology to automate the weighing of a variety of powder samples, which significantly extends the convenience and automation of weighing operations [37]. To adapt to unknown environments and improve the efficiency of the application, the research team also developed a powder weighing simulator, which was used to select the dynamic parameters of DR, enabling the robot to achieve milligram-level accuracy already during the powder weighing process. This approach improves the weighing efficiency during automated experiments. It enhances the generalization ability of the robotic system to meet the challenges of unknown environments, significantly contributing to the automation of chemical experimental processes.

To improve the machine chemist’s ability to recognize routine laboratory containers and the materials in them, Eppel et al. constructed Vector-LabPics, a dataset that annotates containers and the material phases inside them [38]. Neural network models trained on this dataset improved the accuracy of container and material phase recognition. This machine learning-based approach dramatically enhances the machine chemist’s perception and recognition of the laboratory environment.

### 2.3. Intelligent Shared Cloud Synthesis System

With the rapid development of AI, the Internet of Things (IoT), cloud computing, and automation technologies, the overall automation of laboratories has become an important development trend in chemistry and materials research. For example, Roch et al. introduced ChemOS, a software package that connects various types of automation devices in an autonomous experimental platform [39], which covers the core layers required for the operation of autonomous laboratories, including the collection of experimental data, the management of experimental procedures, and the control of related robotic devices, which is characterized by versatility, flexibility, and modularity and significantly reduces the cost of laboratory operation. Meanwhile, the ChemOS software package also supports the remote control of equipment, which enables remote equipment operation between different laboratories and institutions, helping to realize the construction of an intelligent synthesis cloud experimental platform.

**Figure 8:**
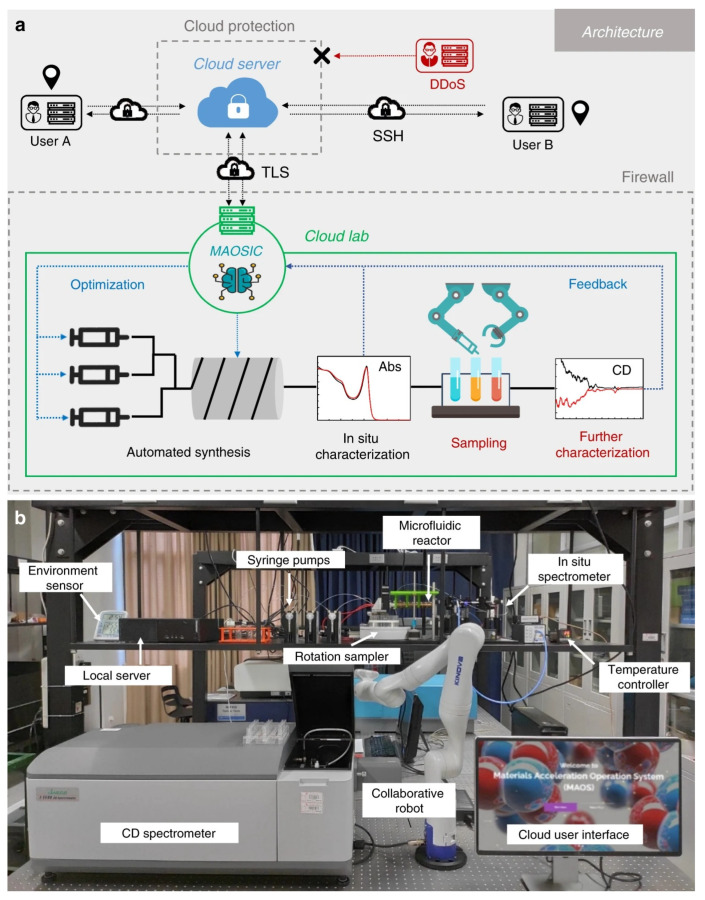
Workflow of cloud lab for discovering new optically active IPNCs. (**a**) MAOSIC allows remote users to interact with (**b**) integrated equipment in lab through cloud server. Encrypted communication and firewalls were utilized to maintain data security and transfer stability. Automatic reactions took place in microfluidic reactors and under in situ monitoring through absorption spectrum. Collaborative robots handled automatic sampling task for CD measurement. All characterization data were sent back to analysis and optimization module in MAOSIC for autonomous optimization. Adapted with permission from ref. [40], copyright 2020, the authors.

Li et al. constructed an intelligent cloud laboratory driven by the materials acceleration operating system in cloud (MAOSIC) platform (Figure 8) [40]. Through the remote collaboration of researchers, optically active inorganic perovskite nanocrystals (IPNCs) were first synthesized with temperature-dependent circular dichroism (CD) and inversion control. Thus, establishing the intelligent synthesis cloud platform reduces the cost of laboratory construction, improves experimental reproducibility and traceability, integrates global resources, improves experimental efficiency, and accelerates the discovery process of new materials.

The advantages of automated synthesis tools in big data analysis, pattern analysis, and precise process control have significantly reduced the requirements for researchers’ operational skills and computational analysis abilities. They are easier for most researchers to master and use. This makes high-throughput computing possible and lowers the threshold for non-professionals to enter the field of computationally assisted material synthesis [41], thus promoting the development and innovation of cross-disciplinary research.

## 3. The Design and Optimization of the Entire Process of Intelligent Synthesis Assisted by Artificial Intelligence

As the level of chemical automation advances, the volume of available experimental data is growing exponentially. Concurrently, improvements in computational power have enabled the widespread application of complex machine learning models in material synthesis [42]. Most machine learning algorithms can infer whether specific prediction, induction, or summarization tasks can be executed based on acquired images, text, or datasets. Moreover, the relationship between parameters in some experimental processes and their outcomes often remains in a “black box” state. In such scenarios, machine learning algorithms prove to be more effective, providing more precise analysis and optimization solutions when augmented by automated synthesis. Additionally, machine learning can guide data collection by identifying the most informative experiments, thereby offering robust support for the accelerated development of efficient nanoparticle synthesis protocols. Machine learning holds promise in facilitating the synthesis of novel nanoparticles with complex properties, potentially driving technological breakthroughs and paradigm shifts in material synthesis [43].

In AI-assisted intelligent research on inorganic nanomaterials, Szymanski et al. proposed a landmark a-lab platform that utilizes large-scale first-principles material phase stability data provided by Materials Project and Google DeepMind [5]. In 17 consecutive days of running experiments, the a-lab platform successfully synthesized 41 new compounds out of 58 targets, and was able to summarize and suggest failed formulations accordingly, thus further improving the success rate of synthesis. This breakthrough research demonstrates the efficiency of AI-based platforms driven by machine learning algorithms, in particular in material discovery, and facilitates further integration of hardware systems, especially robotics.

In the field of intelligent synthesis, the role of AI algorithms becomes increasingly important as the automation of the experimental process increases, accompanied by large amounts of data collection. Machine learning summarizes the published literature and generalizes experimental datasets while optimizing the synthesis process and providing feedback, thus influencing all aspects of inorganic nanomaterial synthesis.

### 3.1. Literature Mining and Analysis

Information gleaned from the scientific literature can help guide and accelerate the development of nanomaterials. However, with the proliferation of the literature and the ever-expanding depth and breadth of the research field, searching the literature and digesting the information can be a time-consuming and manual process for current researchers [44].

This issue consumes researchers’ time and energy while complicating systematic research, especially given the increasing demand for interdisciplinary studies. Therefore, literature mining is essential in intelligent synthesis efforts.

**Figure 9:**
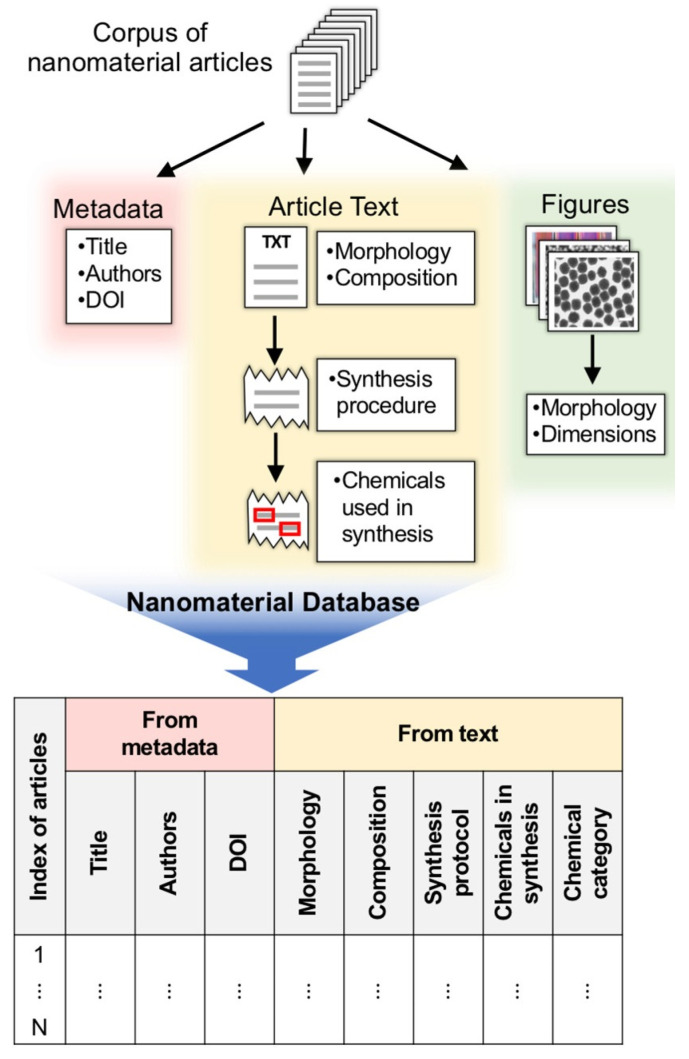
Schematic of processes developed to extract structure information from nanomaterial synthesis articles. Adapted with permission from ref. [45], copyright 2020, American Chemical Society.

Hiszpanski et al. [45] proposed using machine learning to extract structural information from nanomaterial synthesis literature (Figure 9). They developed a scientific literature processing tool that extracts and constructs information from the text and graphs of nanomaterial literature, enabling the creation of personalized nanomaterial synthesis knowledge bases that can be mined to help guide further nanomaterial development [45]. The authors started with a corpus of about 35,000 nanomaterial-related documents, classified the documents according to the composition and morphology of the nanomaterials, extracted the synthesis protocols from the text of the documents, and extracted, normalized, and categorized the chemical terms used in the synthesis protocols. The information obtained through the tool can be used to discern trends in nanomaterial synthesis, such as the correlation of specific reagents with various nanomaterial morphologies, thus helping to guide hypotheses and reduce the space of extraneous parameters during experimental design.

Zhao et al. provided an initial selection of the key synthesis parameters by mining the informative literature related to gold and perovskite NCs to determine the concentration of surfactants for gold nanocrystals (Au NCs) and the type of double perovskite NC active agent [22]. For Au NCs, data mining helped identify the most commonly used surfactants and verified how the parameters of these surfactants affect morphological control. For lead-free double perovskite NCs, data mining was then used to identify potential surfactants and solvents from the literature. In addition, Kim et al. developed a methodological framework that automatically extracts material synthesis parameters from over 12,000 papers using natural language processing techniques [46]. This framework predicts key parameters required for the synthesis of titania nanotubes via hydrothermal methods. It employs machine learning to estimate synthesis outcomes for material systems not included in the training set. The framework demonstrates the capability of migration learning and beyond heuristic strategies, significantly improving the efficiency of data extraction and analysis and providing new strategies for future nanomaterial synthesis research.

Currently, there is an urgent need for researchers to automatically extract synthetic information from massive literature through literature mining systems for the systematic integration and construction of structured databases [47]. However, the field of literature mining still faces challenging problems, such as the difficulty of mining synthetic morphological information in the literature. Therefore, there is an urgent need to develop advanced algorithms and models further to lower the threshold of use for researchers [48]. For example, engineering strategy-guided ChatGPT (GPT-3, GPT-3.5 and GPT-4) can be introduced into nanomaterial synthesis research efforts to enable researchers to conveniently collect information and learn about related research areas [49,50,51], as well as to provide higher-quality data support for machine learning and deep learning models needed for subsequent intelligent synthesis studies of inorganic nanomaterials.

### 3.2. Dataset Construction

In recent years, breakthroughs in experimental techniques and computational methods have significantly increased the scale and complexity of data generation in materials science. In the face of massive raw data with high complexity, efficient storage, and in-depth analysis systems need to be established to exploit their scientific value entirely [52]. Therefore, the construction of specialized databases is of twofold importance: researcher-oriented databases should support the rapid construction and systematic expansion of knowledge frameworks, and algorithm-oriented databases should reduce the dependence on computational resources and the requirements for hardware and software by optimizing the data integration mechanism, thus forming a synergistic research paradigm [45].

From the viewpoint of data source characteristics, existing material synthesis databases can be divided into two categories. One is the literature database, which contains a large number of successful synthesis cases and related experimental data, which are publicly available and easy to retrieve; the other is the experimental database constructed through automated synthesis experiments and a series of iterations, which is mainly used to train machine learning models, focusing on the datasets of specific types of experiments. In terms of data selection, in addition to the successful cases of experimental data, the impact of the breakthrough in arithmetic power should be considered, especially the impact of laboratory data. In addition to successful experimental data, the impact of arithmetic breakthroughs should be fully considered, especially the “hidden” information—i.e., failed or unsuccessful cases—contained in the lab’s archived experimental records. This type of data is also of great value. The inclusion of such data should not be overlooked in the database construction process. In addition, novel databases designed explicitly for constructing predictive synthesis models show good promise [53].

The large number of Au NC and double perovskite NC samples synthesized by the intelligent platform has generated a wealth of characterization and experimental data, allowing the platform to utilize the database for the inverse design of machine learning models [22]. The database includes structure-directing agent (SDA) parameters from the robotic synthesis process, in situ characterization results, and offline validation data. Vaucher et al. constructed a dataset of 693,517 chemical equations and their associated sequences of operations by extracting and processing the text of experimental procedures from patents and analyzing it using an advanced natural language processing model [54]. The trained chemists found that the predicted sequences of operations could be executed without human intervention in more than 50% of the cases.

To overcome the limitation of the lack of a comprehensive database covering the synthesis process, Kononova et al. constructed a “coded recipe” dataset containing 19,488 synthesized entries processed by text mining and natural language processing methods [55]. Each entry includes the target material, starting compound, reaction conditions, and chemical reaction equation. This dataset is publicly available and provides a basis for data mining for the synthesis of inorganic materials.

Gold nanoparticles are highly desirable for applications due to their tunable properties, which depend on the size and shape of the particles. Although many empirical methods have been proposed to control the morphological characteristics of AuNPs, the underlying mechanisms of size and shape control are poorly understood, partly because of the extensive range of combinations of synthesis parameters. With access to sufficient synthesis data, data-driven approaches can provide important help in understanding these mechanisms [56].

To facilitate data mining in nanomaterial synthesis, Cruse et al. constructed and made publicly available a dataset of codified gold nanoparticle synthesis protocols and outcomes extracted directly from the nanomaterial scientific literature through natural language processing and text mining techniques [57]. The dataset contains 5154 data records, each corresponding to a piece of gold nanoparticle synthesis literature that was filtered from 4.97 million publications. Each record contains encoded synthesis protocols as well as morphological information extracted from 7608 experimental passages and 12,519 characterization passages.

The construction of the database is crucial for an intelligent synthesis process. Therefore, it is recommended that data be collected, managed, and shared based on the principles of “findable, accessible, interoperable, and reusable” data. Some self-driven laboratories have accessed open-source databases to reduce experimental costs and improve research efficiency [58]. However, since databases need to be open-sourced to a wide range of researchers, data entry must be carefully screened. Database construction is still in the developmental stage, and input datasets often have human bias and need to be carefully screened to prevent this bias from being perpetuated in machine learning models and affecting prediction accuracy [59]. Effectively constructing databases enhances algorithmic prediction accuracy and inverse design capabilities while improving decision-making accuracy for nanomaterials researchers and boosting the efficiency of human–machine collaboration, thereby advancing materials science [60].

### 3.3. Intelligent Synthesis Scheme Design

#### 3.3.1. Machine Learning-Assisted Synthesis Scheme Design

Machine learning plays an important role in the design of inorganic nanomaterial synthesis schemes [10]. By analyzing a large amount of experimental data and building efficient models, machine learning can effectively analyze the reaction mechanisms and the effects of key parameters on the structure and properties of materials. Since the synthesis of nanomaterials usually involves the regulation of multiple parameters and the complex relationship between these parameters and the resulting products, machine learning can identify the optimal or most suitable synthesis route among the numerous possible routes through the multi-objective optimization method.

Based on experimental data, interpretable machine learning methods, theoretical derivations, and first-principles simulations, Wang et al. developed a theory of metal–oxide interfaces based on metal–metal and metal–oxygen interactions [61], which advances the design of interfaces supporting metal catalysts. In addition, deep learning can significantly improve the efficiency of material discovery by training graph networks on a large scale, showing significant potential, especially in the application of layered materials and solid-state electrolyte candidates [4]. Meanwhile, machine learning methods can predict the binding energies of atoms, molecules, and nanoparticles and the formation energies with practical accuracy [62].

Pre-trained and fine-tuned Large Language Models (LLMs) perform comparably to specially designed machine learning models in predicting the synthesizability of inorganic compounds and selecting precursors and even offer superior performance in some scenarios while also significantly reducing the user expertise, cost, and time required for the development process [63]. This provides researchers with an efficient, low-cost AI tool that contributes to the diffusion of chemical applications. However, it is worth noting that machine learning algorithms cannot be relied upon alone for prediction and summarization. By combining computational methods based on the body of knowledge and experience of chemical experts with machine learning algorithms, the accuracy and success rate of material synthesis can be further improved, especially in terms of providing important support in dealing with rare and specialized reaction types [64].

Jose et al. proposed a materials combination of the Thompson Sampling Efficient Multi-Objective algorithm (TSEMO), the pairing of annular microreactor synthesis, and high-throughput analytical techniques for the material development method to optimize the synthesis process of antimicrobial ZnO nanoparticles successfully [65]. The method constructed an optimization model in less than 100 experiments, achieved a Pareto optimum for synthesis efficiency and material properties, and attained a continuous production capacity of 1 kg per day. The synthesized zinc oxide nanoparticles were equivalent to those obtained by the conventional hydrothermal synthesis method regarding antimicrobial activity. To promote the industrialization of this method, the researchers conducted a scalability assessment, which showed that scaling up the cyclic microreactor by increasing the number of reactors can significantly reduce the complexity of scale-up production.

#### 3.3.2. Optimization of Synthesis Protocols Through Integration of Machine Learning and Microfluidic Technology

Microfluidic technology has been pivotal in designing and optimizing intelligent synthesis schemes for inorganic nanomaterials. Microfluidic technology has achieved a high degree of automation. It is easily integrated with machine learning techniques to realize closed-loop systems, enabling iterative regulation during the synthesis process and enhancing synthesis efficiency. The combination of machine learning and microfluidic technology facilitates the exploration of the relationship between synthesis parameter space and synthesized products, providing significant support for the efficient synthesis of inorganic nanomaterials. Tao et al. studied an autonomous machine learning-driven oscillatory microfluidic platform for the synthesis of AuNPs [66]. This platform integrates various formulations, reaction times, and spectral characterization techniques, utilizing a machine learning model (Gryffin ML algorithm) to analyze spectral data and identify optimal reaction conditions associated with specific optical properties. Gryffin is a machine learning algorithm based on Bayesian optimization, specifically designed for experimental parameter optimization to identify the optimal reaction conditions associated with specific optical properties [67]. Furthermore, the platform allows for an in-depth investigation of the relationship between reaction conditions and the properties of AuNPs. Research indicates that the integration of microfluidic technology with machine learning significantly enhances the efficiency and accuracy of inorganic nanomaterial synthesis.

**Figure 10:**
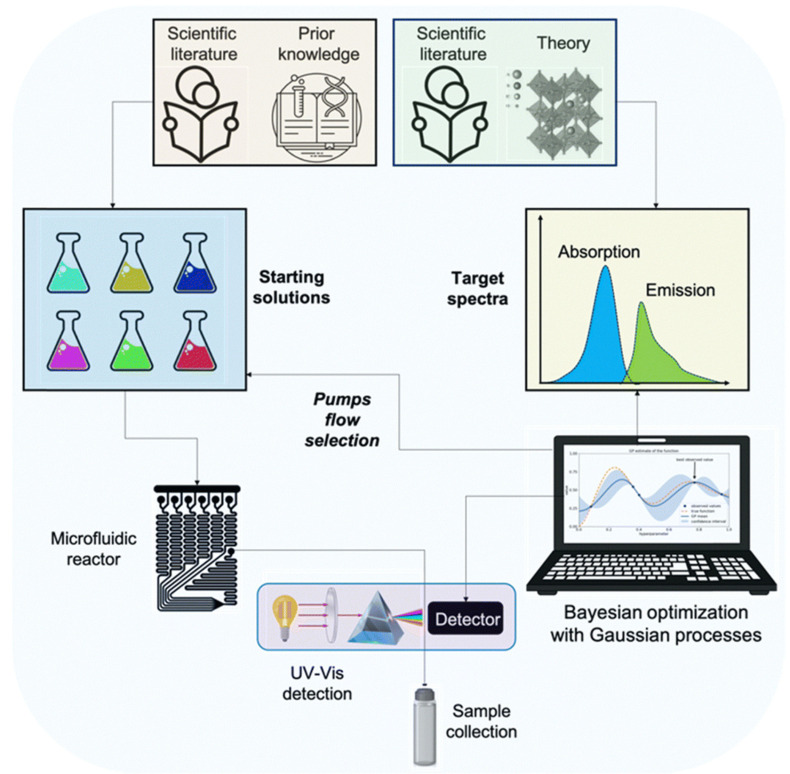
Schematic workflow representation for optimizing synthesis protocol with proposed approach. Adapted with permission from ref. [68], copyright 2024, the authors.

Regarding scalable protocols for machine learning-assisted synthesis, Kioumourtzoglou et al. proposed a novel concept termed “Nanomaterials as a Service” (NaaS), which refers to on-demand batch synthesis protocols for nanomaterials. The platform tailored for this protocol is an automated microfluidic platform based on machine learning (Figure 10) [68]. Users can customize nanomaterials according to their requirements, while manufacturers can achieve the necessary nanomaterials for large-scale production through rapid optimization protocols. This platform combines unsupervised Bayesian optimization with Gaussian processes, effectively shortening optimization times and reducing reliance on prior knowledge. In terms of hardware design, the platform employs polytetrafluoroethylene tubing and fittings, enabling low-cost customized designs. This platform replaces traditional, labor-intensive trial-and-error methods and offers a viable pathway for the standardization and large-scale synthesis of nanomaterials. The platform successfully achieved its bulk production by optimizing the synthesis protocols of silver nanosheets and Prussian blue nanoparticles. This protocol aids in accelerating the translation and commercialization of nanomaterials, making the standardization and scaling of the nanomaterial synthesis process possible.

In summary, integrating microfluidic reactors with machine learning models enhances the research efficiency of novel approaches for synthesizing inorganic nanomaterials and provides strong support for developing new functional materials, particularly in optimizing synthesis pathways. Moreover, applying machine learning to other automated synthesis systems has also significantly contributed to the optimization and design of synthesis protocols.

### 3.4. Intelligent Analysis of Material Characterization Information

In the context of the rapid development of nanoscience, in situ characterization measurement means and tools for nanoparticles have also been rapidly developed [69]. Accurate measurements of nanoparticles are crucial for material property assessment, and the combination of machine learning and nanoscale characterization techniques is expected to improve the efficiency and accuracy of nanomaterial analysis significantly. For example, fast scanning probe microscopy techniques enabled by machine learning can reveal a wide range of nanoscale and time-resolved physical phenomena [70]. Deep learning algorithms can infer key structural features based on material composition in XRD spectrum prediction, enabling downstream predictive analysis [71]. Thanks to the excellent performance of machine learning algorithms in the test set, we can achieve high-throughput screening of the material compositional space through this approach, thereby enhancing the discovery efficiency of target materials.

Due to the diversity and complexity of in situ characterization data of nanomaterials, traditional analysis methods have significant limitations in accuracy and efficiency. The introduction of machine learning effectively improves this problem. The process of in situ characterization and data analysis was comprehensively enhanced by human–machine collaboration to optimize microscope tuning and experiment planning and the optimization capability of machine learning in data analysis [72].

### 3.5. Design of Target-Oriented Retro-Synthetic Pathways

Traditional direct design methods based on material chemistry intuition are often time-consuming and inefficient, making it difficult to meet the future needs of sustainable material discovery. To address this challenge, reverse design has emerged as an important application method in material informatics platforms. This method outputs material solution designs with predefined target properties by extracting potential knowledge from material data.

Machine-driven reverse design strategies can be categorized into three main approaches: high-throughput virtual screening, global optimization, and generative modeling [73,74]. Deep generative modeling has been widely applied to various material classes [3]. Moreover, the approach of exploring inverse synthetic routes by combining Monte Carlo tree searching with symbolic AI techniques has improved efficiency and quality. In double-blind experiments, the quality of the synthetic routes of this method is comparable to that of conventional synthesis methods and is more efficient [2]. The generative model shows high efficiency in exploring the space of effective chemical compositions and can also be used to make crystal structure predictions by learning the distributions of known materials [73].

With the continuous development of machine learning techniques, the concept of inverse design has been extended to the field of inorganic nanomaterials. The electromagnetic enhancement effect triggered by the surface plasmonic quasi-free electron collective oscillations of metallic nanoparticles in the ultraviolet to near-infrared range makes this class of nanoparticles promising for applications in several fields. Usually, the far-field and near-field optical properties of these nanoparticles are obtained by numerical simulations. However, traditional numerical simulations are computationally demanding, and it is difficult to balance accuracy and computational speed. For this reason, He et al. utilized a deep neural network algorithm to establish the mapping between the far-field spectral/near-field distributions and the sizes of three types of plasma nanoparticles (nanospheres, nanorods, and dimers) [75]. The trained deep neural network model was able to accurately and efficiently predict optical properties and nanoparticle size parameters using a screening and resampling approach, allowing the algorithm to complete the near-field prediction in less than 10^2^ s on a laptop computer, which is six orders of magnitude faster than conventional numerical simulations. Voznyy et al. combined machine learning with experimental data to find optimized synthesis parameters of monodispersity of PbS QDs in the space [76], revealing that the introduction of a growth-inhibiting precursor (oleylamine) can lead to nucleation-dominated growth, which leads to the synthesis of large bandgap PbS nanoparticles and optimization of their monodispersity. Wang et al. reported a model-based study on the precise preparation of HgSe QDs and their spectral response [77], revealing the effect of the change in the size of the HgSe QDs on the transmission spectra utilizing both theoretical and experimental modeling and combining the traditional polynomial fitting and machine learning methods to construct a hyperplane model and a neural network model between the synthesis parameters and spectral response of HgSe QDs, realizing the model-based precise preparation of QDs, and demonstrating the effective booster role of machine learning in reverse synthesis and precise preparation.

Machine learning has demonstrated significant advantages in reverse synthesis design, especially in improving efficiency, automated prediction, and optimization of synthesis pathways. With high-throughput screening and global optimization methods, machine learning can significantly improve researchers’ productivity and show substantial advantages in personalizing chemical reaction pathways. However, future research will still require the concerted efforts of interdisciplinary teams to address key issues such as cross-domain knowledge integration, data sharing, and adaptive learning.

Although intelligent synthesis is currently experiencing rapid development and has made significant contributions to various aspects of chemical research, it is also necessary to consider potential issues in its practical applications. One such issue is the reproducibility of AI-generated experimental protocols in industrial applications, which may be compromised due to environmental variations. Therefore, it is essential to exercise strict control over environmental and experimental conditions during the protocol generation process to minimize environmental pollution and resource wastage. This approach ensures a rational and efficient utilization of artificial intelligence in chemical research.

## 4. Typical Cases of Intelligent Synthesis of Inorganic Nanomaterials

The intelligent synthesis system makes the synthesis process of inorganic nanomaterials more efficient, controllable, and reproducible through the precise control of automated equipment. Furthermore, the introduction of algorithms such as machine learning optimizes the reaction routes in real-time during the synthesis process and provides customized solutions for the synthesis of complex materials.

Due to the relatively mature synthesis techniques for quantum dots and gold nanoparticles, and the substantial amount of data available for machine learning algorithms, these materials are particularly suited for intelligent synthesis through the integration of artificial intelligence and automated systems [78,79]. This approach allows for the exploration of parameter space, as their synthesis parameters can significantly influence their properties. Additionally, the characterization methods for these materials are well-suited for use in closed-loop systems, thereby enhancing the efficiency of intelligent synthesis. In addition, typical inorganic nanomaterials such as quantum dots and gold nanoparticles possess unique optical, electrical, and catalytic properties, which are widely applied in various fields, including sensors, drug delivery, and catalytic reactions [80]. Therefore, this paper uses the intelligent synthesis study of representative inorganic nanomaterials, such as QDs and AuNPs, to further elucidate the superiority and convenience of the intelligent synthesis system.

### 4.1. Quantum Dots

In the field of inorganic perovskite quantum dots s(QDs), Epps et al. proposed an “Artificial Chemist” platform, which combines machine learning-based experimental selection with efficient autonomous flow chemistry to synthesize inorganic perovskite QDs successfully [81]. By analyzing the relationship between the reaction conditions and the properties of the QDs, Artificial Chemist synthesized the target QDs and optimized their quantum yields and polydispersity to lie within the target band gap. Notably, the platform demonstrated its strong potential in quantum dot synthesis by rapidly formulating synthesis recipes without any a priori knowledge.

**Figure 11:**
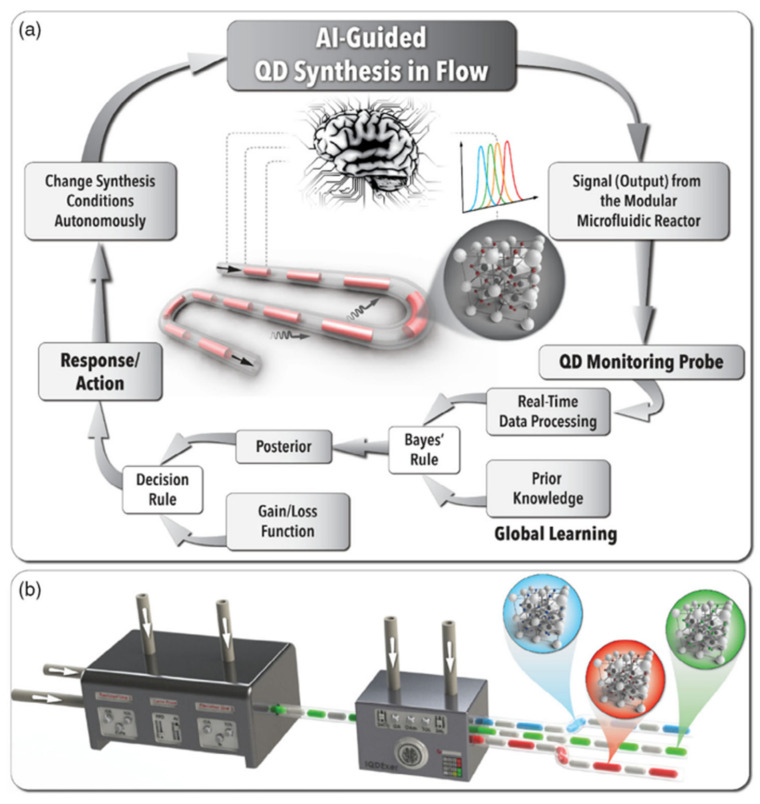
Self-driven multistep quantum dot synthesis enabled by autonomous robotic experimentation in flow. (**a**) Schematic illustration of concept of AI-guided QD synthesis in flow. (**b**) Schematic illustration of on-demand continuous manufacturing of inorganic lead halide perovskite QDs enabled by AI-guided modular microfluidic reactor. Adapted with permission from ref. [82], copyright 2020, the authors.

In another case, Abdel-Latif et al. proposed a modular microfluidic synthesis strategy incorporating an AI-guided decision-making agent to handle complex synthesis parameter spaces. This approach utilizes an autonomous microfluidic experimental strategy to rapidly identify optimal synthesis recipes for perovskite QDs (Figure 11) [82]. This study demonstrates the advantages of combining a microfluidic reactor with a machine learning model, which dramatically improves the efficiency of synthesis research, especially in optimizing reaction pathways, and promotes the discovery and application of novel functional materials.

Metal cation-doped lead halide perovskite QDs exhibit high photoluminescence quantum yields due to quantum cleavage phenomena and are expected to be an important component of next-generation renewable energy technologies. However, the optimization of synthesis routes for lead halide perovskite QDs and the development of high-performance QDs still face many challenges. To address this issue, Bateni et al. proposed a self-driven fluidic lab (SDFL) called Smart Dope [83], which aims to accelerate the exploration and autonomous optimization of the lead halide perovskite QD synthesis space. The lab is modularly constructed, combining a two-phase gas–liquid segmented flow format, a pressure control module, and a miniature spectrometer, enabling the precise control of high temperatures, uniform and stable formation of the reaction liquid, and continuous in situ spectroscopic detection. Through a one-pot synthesis method, SDFL conducted a multi-cation doping study of CsPbCl_3_ QDs. In this experimental platform, the machine learning-assisted decision agent can perform a large number of autonomous experimental operations without human intervention and seamlessly switch to the continuous generation mode after identifying the optimal synthesis conditions, which significantly improves the conversion efficiency from R&D to production. By accelerating the autonomous optimization process, Smart Dope successfully identifies the optimal synthesis conditions for Mn-Yb co-doped CsPbCl_3_ QDs with a photoluminescence quantum yield of 158%. The Smart Dope platform enables closed-loop autonomous experiments by combining the modeling of digital twin quantum dot synthesis with continuous robust experiments and in situ characterization using modern data science tools.

This method significantly enhances the efficiency of discovering and developing renewable energy materials, demonstrating the immense potential of combining machine learning with automated synthesis systems. Moreover, the platform is highly versatile and can be expanded and applied to research on other materials with similar synthesis methods, driving further development in the field.

### 4.2. Gold Nanoparticles

In the intelligent synthesis of AuNPs, Jiang et al. reported in 2022 a synthetic robotic system named AI-EDISON, designed for the exploration, discovery, and optimization of gold nanoparticle structures [84]. This system encompasses a complete iterative cycle, a chemical reaction module, and an integrated experimental platform. AI-EDISON is equipped with a real-time characterization system that combines spectral feedback with machine learning algorithms, enabling the efficient control and optimization of the synthesis process. To enhance the efficiency of synthetic experiments, ensure reproducibility, and facilitate the preservation of experimental samples, the hardware system employs a Geneva wheel structure for its rotating platform, in conjunction with high-precision injection pumps for accurate feeding, sampling, and analysis. Furthermore, essential hardware components are housed within a temperature-controlled chamber to mitigate the impact of environmental variables on the stability of experimental results. The system also features a dedicated seed access device for storing samples synthesized in previous experiments, which can be utilized for subsequent seed induction reactions. This device is equipped with light sources and spectrometers to facilitate real-time characterization and detection.

**Figure 12:**
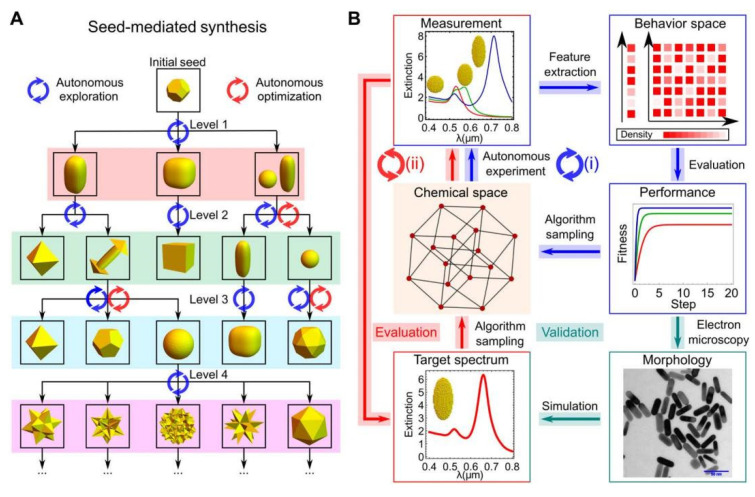
The closed-loop approach toward exploration and optimization in the seed-mediated synthesis of nanoparticles. (**A**) A pictorial representation of AuNPs from hierarchically linked chemical synthetic spaces in the seed-mediated synthesis. (**B**) A closed-loop approach for exploration [(i), blue cycle)] and optimization [(ii), red cycle], respectively. Adapted with permission from ref. [84], copyright 2022, the authors.

In the closed-loop discovery process of AuNPs, the whole process is divided into three main steps (Figure 12): (1) seed-mediated synthesis relying on algorithmic recommendations through liquid dispensing and dynamic pH control; (2) spectroscopic analysis of synthesized products under the recommended conditions, followed by cleaning in preparation for the next synthesis; and (3) the extraction of feature data after real-time characterization, and the optimization and designing of new synthesis conditions. Combined with experimental data and extinction spectroscopy simulations, the method achieved up to 95% yield. The results show that the intelligent synthesis system effectively improves the precision and reproducibility of gold nanoparticle synthesis through closed-loop feedback and algorithmic optimization, providing an advanced tool for the field of material synthesis.

In gold nanoparticle synthesis, machine learning goes beyond optimizing synthesis conditions to integrate natural gene selection optimization methods into its algorithms. By leveraging simulation and data-driven approaches, it assists researchers in discovering novel synthesis pathways. At the same time, the machine learning system can also generate unique digital signatures for the efficient and reproducible synthesis of target materials.

As seen from the above, the intelligent closed-loop synthesis system for QDs and AuNPs demonstrates significant progress in the regularity research of modern nanomaterial science. By integrating automation technology and machine learning algorithms, the intelligent system has remarkably enhanced the efficiency of nanomaterial research and synthesis [85]. During the intelligent synthesis process of QDs, robots utilize machine learning algorithms to optimize synthesis conditions, and explore and predict the optimal experimental parameters, achieving precise control over the synthesis process and thus efficiently obtaining the target products. The intelligent system improves experimental efficiency and effectively reduces the cost of trial-and-error. As a result, its application in the synthesis of inorganic nanomaterials offers researchers new research perspectives [86]. Through automated synthesis, data-driven approaches, and algorithm optimization, the system enhances the efficiency of optimizing synthesis paths for target materials. It lays a solid foundation for discovering new materials.

## 5. Summary and Outlook

In recent years, the rise in “AI+” technologies has injected innovative momentum into the precise synthesis of nanomaterials. Artificial intelligence technologies, exemplified by machine learning and deep learning, have progressively established a new paradigm of synthesis characterized by “data-driven + intelligent optimization” by integrating automated experimental setups with closed-loop feedback systems [56,87,88]. This advancement enhances the efficiency of synthesis and the stability of product quality while also opening up entirely new pathways for the exploration of novel nanomaterials.

This review systematically summarizes the latest trends in the intelligent synthesis of inorganic nanomaterials, highlighting the critical role of intelligent and automated synthesis technologies in advancing the discovery and research of these materials. From the perspective of enhancing both device synthesis efficiency and researchers’ R&D productivity, intelligent synthesis significantly improves experimental efficiency. It reduces research barriers, allowing scientists to focus more on innovative exploration amid vast information. As the complexity of automated synthesis processes continues to increase, the demand for universal synthesis modules for inorganic nanomaterials is gradually rising. Therefore, to achieve the flexible control and precise management of the synthesis process of inorganic nanomaterials, there is an urgent need to establish standardized specifications for specialized and general-purpose equipment modules. Furthermore, by incorporating cutting-edge technologies, such as machine learning, Large Language Models, and digital twins, the operational efficiency and data quality of existing characterization equipment have been significantly improved. It has improved the accuracy of the intelligent synthesis system in aspects such as powder weighing and phase identification of laboratory ware. With the aid of cloud platforms, information related to material synthesis has become globally interconnected, enabling the efficient sharing and utilization of data resources.

The introduction of artificial intelligence into the traditionally researcher-driven complete process of chemical synthesis has greatly improved research efficiency and alleviated the burden on researchers, particularly in areas such as knowledge storage and retrieval. Finally, studies on typical inorganic nanomaterials, such as QDs and AuNPs, within intelligent synthesis systems further validate the optimization role of constructing a complete intelligent closed-loop system in the material discovery process and its significance for the precise control of the synthesis process. Furthermore, significant progress has been made in intelligent synthesis research within the self-assembly of nanoscopic hollow inorganic–organic hybrid material [89], warranting further summarization and exploration in the future [90].

In the era of “AI+”, intelligent and automated synthesis technologies will play an increasingly important role in the field of inorganic nanomaterials [91]. However, research on the intelligent synthesis of inorganic nanomaterials is still facing challenges due to the lack of standardized databases, the difficulty of human–computer synergy, the lack of interdisciplinary integration, and the safety and sustainability of artificial intelligence. Facing these technical bottlenecks, the authors believe that the following directions should be emphasized:

(1) Synergistic development of standardized data resources and model optimization

Since the synthesis process of inorganic nanomaterials involves numerous complex parameters, the existing data have made it challenging to meet the training needs of AI models. The construction of a specialized data resource library and a shared cloud platform for researchers will provide solid data support and resource guarantee for the further development of the intelligent synthesis system. In addition, the development of unified standards for specialized and general equipment will help the construction and promotion of modular experimental platforms and promote the whole field towards standardization and intelligence. Therefore, there is an urgent need to establish a systematic database for inorganic nanosynthesis to solve the bottleneck of model training caused by the existing data dispersion and unstructuredness to realize the optimization of exploring the adaptation of algorithms, such as small sample learning and migration learning, in the prediction of material synthesis.

(2) In-depth exploration of automation and human–machine synergy paradigm

With the increasing degree of automation in laboratory synthesis, the researchers’ understanding of the operation process may be weak. Therefore, while promoting the automation process, it is necessary to pay attention to the inheritance of manual experience and basic experimental skills, explore the new paradigm of human–machine synergy, and give full play to the complementary roles between human innovation ability and the high efficiency of intelligent systems. Human–machine synergy will be an effective approach to conduct scientific research both at present and in the future, that is, the combination of humans’ innovative power and the high efficiency provided by intelligent systems. It is necessary to establish a balanced mechanism between automation and human intervention to prevent cognitive gaps in experiments due to excessive reliance on equipment. This involves developing interpretable interfaces for human–computer interaction and enhancing researchers’ abilities to understand and modify intelligent decision-making logic. In addition, focusing on AI-assisted “counter-intuitive” material design, breaking through the limitations of the traditional trial-and-error method, and developing adaptive synthesis systems with autonomous optimization capabilities are also important ways to promote the innovation of the whole chain of “discovery-verification-application”.

(3) Systematic Breakthroughs in Interdisciplinary Integration

The development of intelligent synthesis systems has raised higher demands for interdisciplinary collaboration, necessitating the organic integration of multiple fields such as materials science, artificial intelligence, automated control, and social sciences. It is essential to further promote interdisciplinary research, accelerate the modular development of standardized cloud experimental platforms, and construct an intelligent synthesis framework that balances technological feasibility with ethical considerations. This framework aims to achieve hardware compatibility expansion and establish an open-source ecosystem for software algorithms.

(4) Ethics Precede Intelligent Synthesis

As the application of artificial intelligence becomes increasingly widespread, it is essential to establish corresponding laws and regulations while enhancing supervision to prevent the misuse of artificial intelligence, which could lead to resource wastage and environmental pollution. Furthermore, it is crucial to develop legal frameworks for the regulation and protection of data generated through artificial intelligence to mitigate data privacy risks. During the development of artificial intelligence, data biases can be addressed by pre-screening data to reduce biases in training models.

In summary, with the continuous advancement of technology, intelligent and automated synthesis techniques are expected to play an increasingly significant role in the field of inorganic nanomaterials. In the future, by establishing a comprehensive data platform, optimizing human–machine collaborative mechanisms, and promoting standardized interdisciplinary cooperation, intelligent synthesis systems are anticipated to lower research barriers further, expand innovation spaces, and enhance the reliability and creativity of material synthesis, thereby injecting new vitality into the field of materials science. However, while applying artificial intelligence, we must remain rational and carefully consider its limitations and potential challenges.

It is foreseeable that, with the ongoing iteration of intelligent synthesis technologies, research on inorganic nanomaterials will gradually achieve a closed-loop paradigm transformation characterized by “precise design–autonomous synthesis–rapid validation”. This process aims to reshape the fundamental paradigms of material development. It may give rise to disruptive material systems across scales and dimensions, providing core material support for strategic fields such as innovative information, new energy, biomedicine, and intelligent manufacturing.

## Data Availability

No new data were created or analyzed in this study. Data sharing is not applicable to this article.

## Abbreviations

The following abbreviations are used in this manuscript:

AI	Artificial intelligence
QDs	Quantum dots
AuNPs	Gold nanoparticles
UV-Vis	Ultraviolet–visible
NCs	Nanocrystals
PTFE	Polytetrafluoroethylene
XRD	X-ray diffraction
TEM	Transmission electron microscopy
LTMR	Low-thermal mass reactor
STEM	Scanning transmission electron microscopy
CAISSe	Cu_1−x_Ag_x_InS_y_Se_1−x_
LSPR	Localized surface plasmon resonance
DR	Domain randomization
IoT	Internet of Things
MAOSIC	Materials acceleration operating system in cloud
IPNCs	Inorganic perovskite nanocrystals
CD	Circular dichroism
Au NCs	Gold nanocrystals
SDAs	Structure-directing agents
LLMs	Large Language Models
TSEMO	Thompson Sampling Efficient Multi-Objective algorithm
NaaS	Nanomaterials as a Service
SDFL	Self-driven fluidic lab
